# Presence of Round Cells Proteins do not Interfere with Identification of Human Sperm Proteins from Frozen Semen Samples by LC-MS/MS

**DOI:** 10.3390/ijms20020314

**Published:** 2019-01-14

**Authors:** Manesh Kumar Panner Selvam, Ashok Agarwal, Tânia R. Dias, Ana D. Martins, Luna Samanta

**Affiliations:** 1 American Center for Reproductive Medicine, Cleveland Clinic, Cleveland, OH 44195, USA; manesh.balu@gmail.com (M.K.P.S.); taniadias89@gmail.com (T.R.D.); anacdmartins@gmail.com (A.D.M.); lsamanta@ravenshawuniversity.ac.in (L.S.); 2 Universidade da Beira Interior, 6201-001 Covilhã, Portugal; 3 Department of Microscopy, Laboratory of Cell Biology, Institute of Biomedical Sciences Abel Salazar and Unit for Multidisciplinary Research in Biomedicine, University of Porto, 4050-313 Porto, Portugal; 4 Redox Biology Laboratory, Department of Zoology, School of Life Sciences, Ravenshaw University, Cuttack 753003, India

**Keywords:** sperm, leukocytes, round cells, non-spermatogenic cells, frozen semen, proteomics

## Abstract

In sperm proteomic experiments round cells and leukocyte proteins are profiled along with sperm proteome. The influence of round cell and leukocyte proteins on the sperm proteome has not been investigated. The objective of this study was to identify if the proteins from round cells, including leukocytes, interfere with the proteomic analysis of spermatozoa in frozen semen samples. Proteomic profiling of sperm was performed using liquid chromatography-tandem mass spectrometry in four groups: Group 1 contained neat semen with round cells and leukocytes ≥ 1 × 10^6^/mL, group 2 contained neat semen with round cells ≥ 1 × 10^6^/mL that was processed by 65% density gradient to remove the round cells and leukocytes, group 3 contained neat semen with round cells < 1 × 10^6^/mL, and group 4 contained neat semen with round cells < 1 × 10^6^/mL that was processed by 65% density gradient to remove the round cells. Pure leukocyte culture was used as control group. A total of 1638, 1393, 1755, and 1404 proteins were identified in groups 1, 2, 3, and 4, respectively. Comparative analysis of group 1 vs. 3 revealed 26 (1.18%) differentially expressed proteins (DEPs). On the other hand, only 6 (0.31%) DEPs were observed with group 2 vs. 4. Expression of these DEPs were either absent or very low in the control group. The results of our proteomics analysis failed to show any influence of non-spermatogenic round cell proteins on sperm proteome identification. These results validate the use of neat semen samples for sperm proteomic studies.

## 1. Introduction

Seminal ejaculate is composed of secretions from testis (5%) and other accessory sex glands, such as seminal vesicles (50–65%), prostate (20–30%), and bulbourethral glands (<5%) [1,2]. The main cellular portion of the semen is composed by spermatozoa. However, semen also contains non-sperm cells known as round cells, either of spermatogenic or non-spermatogenic origin [3]. Spermatogenic round cells include immature germ cells and degenerated spermatids, whereas non-spermatogenic round cells include exfoliating epithelial cells form prostate and seminal vesicles and inflammatory cells, such as leukocytes, lymphocytes, and macrophages [4,5]. Basic semen analysis is performed on neat semen samples and includes the estimation of round cell concentrations. When round cells are present in excessive amounts (≥1 × 10^6^/mL), the Endtz test or peroxidase staining test is used to detect the leukocyte population [6,7]. 

The presence of leukocytes ≥ 1 × 10^6^/mL is an indication of genital tract infection or inflammation [8]. The level of seminal leukocytes are elevated in recurrent cigarette smoking and alcohol consuming men [9,10]. These seminal leukocytes are high producers of reactive oxygen species (ROS), which are harmful for spermatozoa when present in excessive amounts, as they induce oxidative stress. ROS can cause nuclear and mitochondrial DNA damages, thus significantly affect sperm function and motility [11]. To minimize the ROS levels, semen samples can be subjected to density gradient centrifugation to remove the seminal leukocytes [12,13,14].

In the era of omics, sperm proteins constitute a great source for understanding the molecular mechanisms of sperm function. Mass spectrometry is a commonly used technique for quantitative proteomic studies [15]. It provides the basis for understanding the biological pathways regulating the normal physiological function of spermatozoa. Sperm proteomic analysis starts with the isolation and separation of sperm cells from seminal plasma. For proteomic experiments, semen samples are frequently stored at −80 °C. However, while freezing the neat semen samples, spermatozoa are stored along with round cells and debris. When the frozen samples are thawed, the round cells present in semen burst. Hence, the extracted total protein will contain a mixture of both sperm and round cell proteins [16]. Gradient centrifugation techniques are widely used to process the semen samples to obtain a pure population of spermatozoa. To date, some studies have used only pure cultures of sperm (without round cells) for global proteomic analysis [17,18,19,20,21], whereas others have used neat semen samples [20,22,23,24]. Thus, it is still unclear (1) whether the processing of semen samples have any interference in the sperm protein expression, and (2) what the contribution is of non-spermatogenic proteins in the evaluation of sperm proteomic profile. The objective of this study was to evaluate the impact of purification (by density gradient) on the sperm proteome detected by liquid chromatography-tandem mass spectrometry (LC-MS/MS) technique. 

## 2. Results

### 2.1. Semen Parameters

Sperm concentration (81.88 ± 45.51 × 10^6^/mL) and motility (69.79 ± 10.02%) were within normal reference ranges defined by the 2010 WHO guidelines [25]. In the samples used for proteomic analysis, the average leukocyte concentration in leukocytospermic samples (*n* = 5) was 1.30 ± 0.57 × 10^6^/mL, while the average round cell concentration for samples (*n* = 5) with round cells < 1 × 10^6^/mL was 0.50 ± 0.26 × 10^6^/mL ( Supplementary Table S1).

### 2.2. Proteomic Profile of Spermatozoa in Different Groups

**Figure 1:**
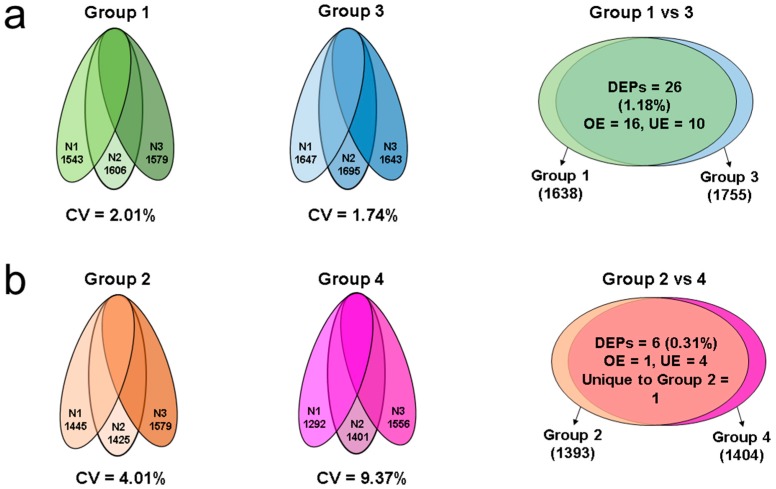
Comparative proteomic analysis of semen samples with round cells and leukocytes ≥1 × 106/mL or round cells < 1 × 10^6^/mL. (**a**) Total number of proteins detected in group 1 and group 3 run in triplicate (N1, N2, and N3), and differentially expressed proteins in group 1 vs. group 3; and (b) total number of proteins detected in group 2 and group 4 run in triplicate (N1, N2, and N3), and differentially expressed proteins in group 2 vs. group 4. Group 1 contained neat semen with round cells and leukocytes ≥ 1 × 10^6^/mL, group 2 contained neat semen with round cells ≥ 1 × 10^6^/mL that was processed by 65% density gradient to remove the round cells and leukocytes, group 3 contained neat semen with round cells < 1 × 10^6^/mL, and group 4 contained neat semen with round cells < 1 × 10^6^/mL that was processed by 65% density gradient to remove the round cells. CV: coefficient of variation, DEPs: differentially expressed proteins, OE: overexpressed, UE: underexpressed.

LC-MS/MS analysis of pooled samples from group 1, 2, 3, and 4 resulted in a total of 1638, 1393, 1755, and 1404 proteins, respectively. A total of 1486 proteins were identified in control group (leukocyte pure culture). Variation in the total number of proteins identified for triplicate runs were 2.01%, 4.01%, 1.74%, and 9.37% in group 1, 2, 3, and 4, respectively (Figure 1a,b, Supplementary Table S2). Comparative analysis of sperm proteins in group 1 vs. 3 (neat semen) revealed a total of 26 (1.18%) DEPs. Among them, 16 DEPs were overexpressed and 10 DEPs were underexpressed in group 3 (Figure 1a). When comparing group 2 vs. 4 (65% gradient processed semen), only six (0.31%) DEPs were identified (Figure 1b). In group 4, four proteins were underexpressed (AKAP3: A-kinase anchor protein 3, CLPTM1: cleft lip and palate transmembrane protein 1, SLC25A15: mitochondrial ornithine transporter 1, and FAM210A: protein FAM210A), the mitochondrial protein cytochrome c oxidase subunit 6A1 was overexpressed, and monocarboxylate transporter 10 (SLC16A10) was uniquely expressed in group 2. The DEPs differed in their abundance in all the four different groups (Table 1 and Table 2). Lists of all the proteins detected in group 1, 2, 3, and 4 and control group are provided in Supplementary Table S3.

### 2.3. Comparative Analysis of Human Sperm Protein and Leukocyte Protein

Proteomic profile of group 1, group 3, and control group revealed only 11 DEPs were identified in all three groups. Key proteins associated with sperm function (IZUMO1, PSMD3) were absent and ATP1A1 was underexpressed in control group (Table 3). 

### 2.4. Molecular Pathways Regulated by DEPs in Spermatozoa

**Figure 2:**
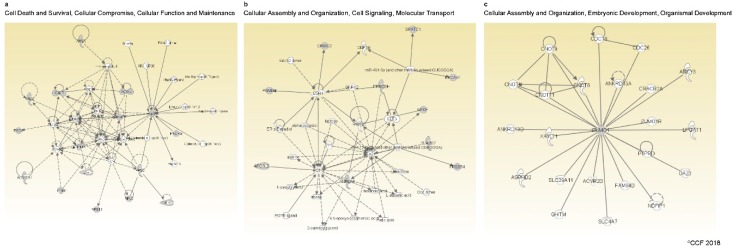
Top networks associated with differentially expressed proteins in group 1 vs. group 3. (**a**) Cell death and survival, cellular compromise, cellular function, and maintenance; (**b**) cellular assembly and organization, cell signaling, molecular transport; and (**c**) cellular assembly and organization, embryonic development, organismal development. Group 1: neat semen samples with round cells and leukocytes ≥ 1 × 10^6^/mL. Group 3: neat semen samples with round cells < 1 × 10^6^/mL.

Networks generated for DEPs between group 1 and 3 using IPA software revealed that 15 proteins were involved in the cell death and survival, cellular compromise, cellular function, and maintenance pathways (Figure 2a). Whereas aberrant expression of 10 proteins was associated with processes such as cellular assembly and organization, cell signaling, and molecular transport in the spermatozoa (Figure 2b). Functional network analysis also identified Izumo sperm-egg fusion protein 1 (IZUMO1) as a key molecule regulating the cellular assembly and organization, embryonic development, and organismal development pathways (Figure 2c).

## 3. Discussion

Cellular and molecular functions of a cell are regulated by proteins. In proteomics, selection of a suitable sample, protein extraction technique, and contaminant removal methods are very critical. Transcriptionally and translationally silent spermatozoa depend on the proteins and their post-translational modifications in order to carry out their normal physiological functions to ultimately fertilize the oocyte [26,27]. Therefore, accurate identification and quantification of proteins in mature and immature spermatozoa provides insight into the function of each protein. Concentration of proteins varies from sample to sample, depending on the yield of the spermatozoa and other cells co-existing with sperm cells. In semen, spermatozoa are present along with other non-sperm cells, especially round cells of spermatogenic and non-spermatogenic origin [3,4,5]. In general, prior to the use of semen samples in proteomic experiments, they are stored as whole ejaculate at −80 °C. However, it remains to be elucidated what the contribution is of the non-spermatogenic proteins to the evaluation of sperm proteome. 

In this study, the intra-assay variation for group 2 and 4 was 4.01% and 9.37%, respectively (Figure 2b). We have identified that only six proteins were differentially expressed between group 2 and 4 (Table 1). This indicates that the number of DEPs identified is very negligible (0.31%) compared with the total number of proteins identified in each group individually (Figure 2b). The majority of the proteins (AKAP3, CLPTM1, SLC25A15, and FAM210A) were underexpressed in group 4, while proteins SLC16A10 and COX6A1 were overexpressed (Table 1). 

Functional annotation of the identified DEPs revealed that the proteins AKAP3 and COX6A1 are associated with sperm function. AKAP3 tyrosine phosphorylation levels regulates the degradation of AKAP3, which in turn is essential for the sperm capacitation process [28]. It is exclusively present in the principal piece of the flagellum of mature spermatozoa and is also expressed in elongated spermatids [29]. In the present study, AKAP3 was underexpressed in group 4 compared to group 2, suggesting the predominant existence of immature spermatozoa in semen samples with round cells and leukocytes > 1 × 10^6^/mL. Mitochondrial protein COX6A1 plays a protective role during the excessive production of ROS [30]. Downregulation of COX6A1 in group 2 shows the presence of abnormal spermatozoa (with mitochondrial dysfunction) compared to group 4. Overall, the difference in the expression of proteins associated with sperm function, along with other proteins, are mainly due to the presence of abnormal spermatozoa in leukocytospermic samples. Therefore, despite the use of density gradient centrifugation to separate sperm population from non-sperm cells, the protein profile of normal spermatozoa from two different methods does not match exactly. This is mainly because of the presence of spermatozoa with varying maturation levels in the semen [31]. 

We also compared the sperm protein profile of frozen neat semen samples containing round cells and leukocytes ≥ 1 × 10^6^/mL (group 1) with samples having round cells < 1 × 10^6^/mL (group 3). A total of 26 proteins (1.18%) were differentially expressed between group 1 and 3 (Figure 2a). These DEPs were less than the intra-assay variability observed in group 1 (2.01%) and group 3 (1.74%). Bioinformatic analysis identified the top networks in which these DEPs were involved in cellular functions, such as (i) cell death and survival, cellular compromise, cellular function, and maintenance, and (ii) cellular assembly and organization, cell signaling, and molecular transport. 

From the top networks, we noticed the overexpression of sperm specific proteins, such as ARSA, ALDH1A1, lactotransferrin (LTF), and calreticulin (CALR). ALDH1A1 is an indicator for impaired spermatogenesis or generation of abnormal sperm cells [32]. Overexpression of ALDH1 in group 1 suggest the higher abundance of spermatogenic round cells. Similarly, CALR protein is involved in the spermatogenic process and fertility potential [33]. Our proteomic analysis data show an overexpression of these proteins, which indicate the higher presence of abnormal spermatogenic round cells in group 1 relative to group 3. The protein ARSA is involved in fertilization and interacts with the heat shock 70 KDa protein 2 (HSPA2) and sperm adhesion molecule 1 (SPAM1), mediating sperm-egg interaction [34]. ARSA is also localized in acrosomal region of round and elongated spermatids [35]. In our study, the overexpression of ARSA in group 1 suggests higher presence of round spermatids and immature spermatozoa compared to group 3. Moreover, the protein LTF, localized with the eppin protein complex on the surface of spermatozoa [36], was overexpressed in group 1 (Table 2). It is abundantly expressed in seminal plasma samples with increased ROS levels [37]. Our LC-MS/MS results are in agreement with earlier reports suggesting that LTF is a sperm-specific protein. The identified networks suggest that these DEPs play an important role in spermatogenesis and maturation of spermatozoa, and alterations in their expression may compromise those mechanisms. This is substantiated by the presence of excessive round cells, including leukocytes in group 1, when compared with group 3. Expression of these proteins are either absent or very low in the control group (pure leukocyte culture) (Table 3). Thus, non-spermatogenic protein contribution to the whole sperm proteome is not significant. 

Network analysis also revealed IZUMO1 as a focus molecule (Figure 2c), which was overexpressed in group 3. IZUMO1 is a membrane protein highly specific to spermatozoa and essential for spermatozoa fusion with the oocyte [38,39]. Likewise, isoform 4 of sodium/potassium-transporting ATPase subunit alpha-1 (ATP1A1) was also overexpressed in group 3 and it is essential for sperm capacitation [40,41]. Another overexpressed protein was the 26S proteasome non-ATPase regulatory subunit 3 (PSMD3), a component of ubiquitin-proteasome pathway, which along with proteasome machinery, regulates the exocytosis of the acrosome during fertilization [42,43]. The overexpression of IZUMO1, ATP1A1, and PSMD3 represents a sign of highly functional spermatozoa in group 3 relative to group 1. Therefore, the differential expression of proteins associated with sperm function between these groups is mainly due to the presence of abnormal sperm population in group 1 compared to group 3. Absence of IZUMO1 and PSMD3 proteins in leukocytes pure culture and significant expression of ATP1A1 in sperm samples (Table 3) indicates that the interference of non-spermatogenic proteins with sperm proteome is very negligible. 

Apart from the DEPs associated with sperm function, the remaining DEPs observed in group 1 when compared with group 3 were either absent or significantly expressed in spermatozoa (Table 3). The functional pathways in which they are involved are of testicular or germ cell origin, and are not due to contamination of leukocytes or somatic cells. Therefore, the question of proteome contamination from other somatic round cells, including leukocytes, present in the semen is ruled out. Henceforth, the proteome of the samples containing round cells is rich in sperm proteins and proteins of non-spermatogenic origin were masked by sperm proteins. Thus, the purification of spermatozoa by density gradient is not essential for the accurate proteomic and bioinformatic analysis of sperm proteins.

This is the first study in the field of sperm proteomics that demonstrates the lack of interference of non-spermatogenic proteins in frozen semen samples. We validate the use of neat semen samples for sperm proteomic studies by LC-MS/MS. Further studies should examine the role of proteins in the immature germ cells (including spermatogenic round cells), aside from the known functions of proteins in mature and healthy sperm.

## 4. Materials and Methods

### 4.1. Semen Analysis

This study was approved by the Institutional Review Board of Cleveland Clinic (Cleveland, OH, USA). A total of 14 donors (normozoospermic; *n* = 14) enrolled in the study were provided with written consent. Semen samples were collected in a sterile container after a minimum of 48 h sexual abstinence. The samples were liquefied in an incubator at 37 °C for 30 mins. After complete liquefaction, macroscopic semen parameters, such as volume, color, pH, and viscosity, were measured. Viscosity of hyperviscous samples was broken down mechanically by repeated pipetting. The use of proteolytic enzymes was avoided for viscosity treatment as it interferes in proteomic analysis [44]. Microscopic semen parameters including sperm concentration, motility, and presence of round cells were determined according to World Health Organization (WHO) guidelines [25]. Additionally, Endtz test was conducted to measure leukocyte concentration (peroxidase positive cells) in semen samples when round cells ≥ 1 × 10^6^/mL [7]. The semen specimens were first divided into (1) samples with round cells ≥1 × 10^6^/mL and leukocytes ≥1 × 10^6^/mL, and (2) samples with round cells < 1 × 10^6^/mL. 

### 4.2. Inclusion and Exclusion Criteria

Fertility status of the volunteers was not considered for including study subjects. Semen samples with more than 1.5 mL in volume were included, while samples with agglutination were excluded from the study. 

### 4.3. Processing of Semen Samples

**Figure 3:**
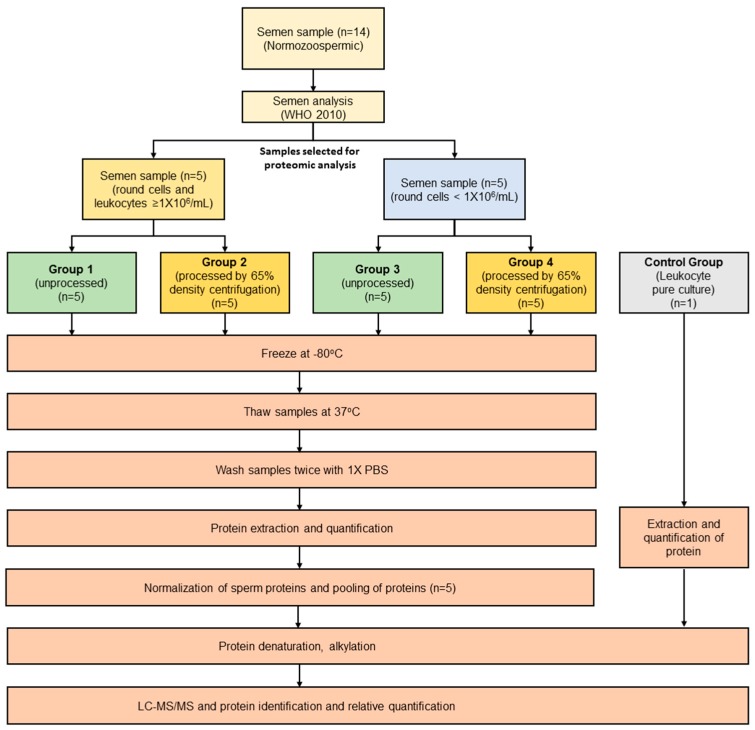
Schematic flowchart of overall experimental design. Normozoospermic samples were divided into four groups. Proteomic profile of sperm was evaluated by LC-MS/MS and compared with a pure leukocyte culture.

Each sample was divided into two equal aliquots. One of the aliquots was immediately stored as unprocessed (neat semen) at −80 °C. The other aliquot was processed by 65% single gradient centrifugation (Figure 3). Briefly, the liquefied neat semen was overlaid on the 65% gradient solution and centrifuged at 300× *g* for 20 min. The seminal plasma was removed and discarded. The white buffy coat ring (containing round cells, including leukocytes, and debris) formed between the seminal plasma and the gradient was also discarded. Furthermore, the gradient containing spermatozoa was diluted in phosphate buffered saline (PBS) and centrifuged at 3000× *g* for 15 min. The sperm pellet free from any round cells was stored at −80 °C.

### 4.4. Experimental Groups

Based on the concentration of round cells and leukocytes, and the sample processing, the experimental groups were defined as follows: Group 1 contained neat semen with round cells and leukocytes ≥ 1 × 10^6^/mL; group 2 contained neat semen with round cells ≥ 1 × 10^6^/mL that was processed by 65% density gradient (PureCeption, CooperSurgical Fertility and Genomic Solutions, Copenhagen, Denmark) to remove the round cells and leukocytes; group 3 contained neat semen with round cells < 1 × 10^6^/mL; group 4 contained neat semen with round cells < 1 × 10^6^/mL that was processed by 65% density gradient to remove the round cells. We have also included a human leukocyte pure culture (Innovative Research, Novi, MI, USA) as control group.

### 4.5. Preparation of Samples for Proteomic Analysis

Frozen samples were thawed at 37 °C and centrifuged at 3000× *g* for 10 min. Sperm pellet was resuspended and washed twice with PBS. The radio-immunoprecipitation assay (RIPA) lysis buffer (Sigma-Aldrich, St. Louis, MO, USA) supplemented with the proteinase inhibitor cocktail (Roche, Indianapolis, IN, USA) was added to the sperm pellet (approximately 100 µL/100 × 10^6^ spermatozoa). Samples were incubated overnight at 4 °C for a complete cell lysis. Then, samples were centrifuged at 13000× *g* for 20 min, the supernatant was aspirated to a new vial, and protein concentration was determined using the bicinchoninic acid (BCA) kit (Thermo, Rockford, IL, USA), according to the manufacturer’s instructions. The same protocol was adapted for extraction of proteins from the leukocyte pellet. 

### 4.6. Quantitative Proteomic Analysis

Five sperm samples from each experimental group (1, 2, 3, and 4) were subjected to proteomic analysis by LC-MS/MS for maintaining biological variability. The same amount of protein was used from each individual sample to normalize the total protein concentration in each group. Sperm samples (*n* = 5) were pooled to create each treatment. Pooling of samples for sperm proteomic analysis is a common practice and is reported in previous reports [22,37,45,46,47,48]. All the protein samples (~30 µg/sample) were run in triplicate (N1, N2, and N3) in 1D-PAGE to maintain technical variability. After completion of electrophoresis, each gel lane was cut into 6 pieces. The samples (cut pieces) were alkylated with iodoacetamine and reduced with dithiothreitol. In-gel digestion was carried out using 5 μL trypsin (10 ng/μL) and 50 mM ammonium bicarbonate, and incubated overnight at room temperature. Peptides from the digested gel were extracted using acetonitrile (50%) with formic acid (5%). Finally, samples were diluted with 1% acetic acid and used for LC-MS/MS analysis.

### 4.7. Liquid Chromatography-Tandem Mass Spectrometry (LC-MS/MS) Analysis

Firstly, samples (5 μL) of the peptide solutions were injected into high performance liquid chromatography (Dionex 15 cm × 75 μm, id Acclaim Pepmap C18, 2 μm, 100 Å reversed phase capillary chromatography) column. Fractions containing the peptides were eluted in acetonitrile/0.1% formic acid at a flow rate of 0.25 μL/min. Each peptide fraction was introduced into the source of Finnigan LTQ-Orbitrap Elite hybrid mass spectrometer on-line. The micro electrospray ion source was operated at 2.5 kV. Data dependent multitask ability of the instrument was used to complete a full spectral scan and to determine molecular weight and amino acid sequence of the peptides [37].

### 4.8. Database Searching

Proteome Discoverer version 1.4.1.288 was used to extract tandem mass spectra. Mascot (Matrix Science, London, UK; version 2.3.02), Sequest (XCorr Only) (Thermo Fisher Scientific, San Jose, CA, USA; version IseNode in Proteome Discoverer 2.2.0.388) and X! Tandem (The Global Proteome Machine (GPM), thegpm.org; version CYCLONE (2010.12.01.1)) were used in data searching process for all the samples. Mascot and X! Tandem were set up to search the human SwissProtKB database (July 2017 version, 42210 entries), assuming the digestion enzyme trypsin. Sequest (XCorr Only) was set up to search Human_SwissProt_July2017.fasta (42152 entries), also assuming trypsin. Carbamidomethyl of cysteine was specified as a fixed modification and oxidation of methionine was specified as a variable modification. Additional settings for data searches include fragment ion mass tolerance of 0.60 Da and a parent ion tolerance of 10.0 PPM. Results of a Mascot search were filtered using a threshold peptide ion score of 40 and at least two matching peptides. For the Sequest searches, the results were filtered based on the XCorr scores < 1.5 (+1 ions), > 2.0 (+2 ions), >2.5 (+3 ions). The false discovery rate (FDR) for these searches was determined to be less than 1%. 

### 4.9. Criteria for Protein Identification

Identified peptides and proteins were validated using Scaffold (version Scaffold_4.8.6, Proteome Software Inc., Portland, OR, USA). With the help of Scaffold delta-mass correction, peptide identifications were accepted if they could be established at greater than 95.0% probability by the Peptide Prophet algorithm [49]. For proteins, the probability had to be >90% to achieve an FDR less than 1% and contained at least 2 identified peptides. Protein probabilities were assigned by the Protein Prophet algorithm [50]. Proteins that contained similar peptides and could not be differentiated based on MS/MS analysis alone were grouped to satisfy the principles of parsimony.

### 4.10. Comparative Proteomics and Identification of Differentially Expressed Proteins

The proteomic profile of all groups were analyzed. Coefficient of variation was used to measure intra assay variability (between the technical replicates) for group 1, 2, 3, and 4. Comparative analysis of the DEPs were performed between group 1 and group 3; and group 2 and group 4. Statistical significance of proteomic results was assessed by independent *t*-test. Different *p*-values were considered according to the abundance of the proteins: (i) Very Low abundance: spectral count range 1.7–7, *p* ≤ 0.001, and NSAF ratio ≥ 2.5 for overexpressed, ≤0.4 for underexpressed proteins; (ii) low abundance: spectral count range 8–19, *p* ≤ 0.01, and NSAF ratio ≥ 2.5 for overexpressed, ≤0.4 for underexpressed proteins; (iii) medium abundance: spectral count range between 20 and 79, *p* ≤ 0.05, and NSAF ratio ≥ 2.0 for overexpressed, ≤0.5 for underexpressed proteins; (iv) high abundance: spectral counts > 80, *p* ≤ 0.05, and NSAF ratio ≥ 1.5 for overexpressed, ≤ 0.67 for underexpressed proteins.

### 4.11. Bioinformatic Analysis of DEPs

Annotation of proteins was performed using Gene Ontology (GO) terms from National Center for Biotechnology Information (NCBI). Also, protein IDs and symbols were extracted from uniport database. DEPs identified in both study groups were subjected to functional annotation and enrichment analysis using publicly available bioinformatic annotation tools and databases, such as Universal Protein Resource (UniProt), Reactome and Database for Annotation Visualization and Integrated Discovery (DAVID) (http://david.niaid.nih.gov), and proprietary curated database Ingenuity pathway analysis (IPA) to analyze the involvement of DEPs in biological and cellular processes, pathways, regulatory networks, and protein-protein interactions.

## Figures and Tables

**Table 1:** Differentially expressed proteins and their abundance in semen samples from group 1 (≥1 × 10^6^/mL leukocytes) and group 3 (round cells < 1 × 10^6^/mL).

Uniprot No.	Gene Name	Protein Name	Group 1		Group 3		NSAF Ratio	Expression
			SC	Abun	SC	Abun		
P15289	ARSA	Arylsulfatase A	34.0	M	1.7	VL	0.05	UE
P00352	ALDH1A1	Retinal dehydrogenase 1	26.3	M	2.7	VL	0.09	UE
Q6PEW0	PRSS54	Inactive serine protease 54	18.0	L	4.7	VL	0.22	UE
Q76KD6	SPATC1	Speriolin	8.3	L	2.3	VL	0.26	UE
Q9HAE3	EFCAB1	EF-hand calcium-binding domain-containing protein 1	9.3	L	3.3	VL	0.33	UE
O43707	ACTN4	Alpha-actinin-4	44.7	M	23.3	L	0.47	UE
P02788	LTF	Lactotransferrin	2486.7	H	1299.0	H	0.47	UE
P14314	PRKCSH	Glucosidase 2 subunit beta	123.0	H	65.0	M	0.48	UE
P27797	CALR	Calreticulin	271.7	H	175.0	H	0.57	UE
P14625	HSP90B1	Endoplasmin	987.0	H	660.3	H	0.60	UE
P05023-4	ATP1A1	Isoform 4 of Sodium/potassium-transporting ATPase subunit alpha-1	98.7	H	181.3	H	1.61	OE
O95202	LETM1	Mitochondrial proton/calcium exchanger protein	29.0	M	65.7	M	2.04	OE
O43242	PSMD3	26S proteasome non-ATPase regulatory subunit 3	33.7	M	78.3	M	2.04	OE
P23396	RPS3	40S ribosomal protein S3	24.7	M	60.0	M	2.20	OE
P11234	RALB	Ras-related protein Ral-B	9.0	L	22.0	M	2.25	OE
Q9NZM1-3	MYOF	Isoform 3 of Myoferlin	21.3	M	55.3	M	2.27	OE
Q9Y4W6	AFG3L2	AFG3-like protein 2	15.3	L	40.7	M	2.38	OE
Q96RQ1	ERGIC2	Endoplasmic reticulum-Golgi intermediate compartment protein 2	8.3	L	23.0	M	2.52	OE
Q8IYV9	IZUMO1	Izumo sperm-egg fusion protein 1	9.0	L	28.7	M	3.00	OE
Q04609	FOLH1	Glutamate carboxypeptidase 2	27.0	M	96.3	H	3.21	OE
Q8IY17-4	PNPLA6	Isoform 4 of Neuropathy target esterase	3.7	VL	14.0	L	3.36	OE
P62277	RPS13	40S ribosomal protein S13	5.3	VL	20.7	M	3.42	OE
P46777	RPL5	60S ribosomal protein L5	5.7	VL	24.7	M	3.93	OE
Q13093	PLA2G7	Platelet-activating factor acetylhydrolase	5.3	VL	22.7	M	4.02	OE
O43653	PSCA	Prostate stem cell antigen	1.7	VL	14.3	L	7.22	OE
Q96M98-2	PACRG	Isoform 2 of Parkin coregulated gene protein	1.3	VL	21.0	M	13.04	OE

VL: very low; L: low; M: medium; H: high; UE: underexpressed; OE: overexpressed; NSAF: normalized spectral abundance factor; Abun: abundance; SC: spectral count.

**Table 2:** Differentially expressed proteins and their abundance in processed semen samples from group 2 (≥1 × 10^6^/mL leukocytes) and group 4 (round cells < 1 × 10^6^/mL).

Uniprot No.	Gene Name	Protein Name	Group 2		Group 4		NSAF Ratio	Expression
			SC	Abun	SC	Abun		
O75969	AKAP3	A-kinase anchor protein 3	146.3	H	81.7	H	0.56	UE
O96005	CLPTM1	Cleft lip and palate transmembrane protein 1	41.3	M	19.0	L	0.45	UE
Q9Y619	SLC25A15	Mitochondrial ornithine transporter 1	26.0	M	12.0	L	0.47	UE
Q8TF71	SLC16A10	Monocarboxylate transporter 10	2.0	VL	0.0	-	0.00	Unique to Group 2
Q96ND0	FAM210A	Protein FAM210A	5.0	VL	0.7	VL	0.14	OE
P12074	COX6A1	Cytochrome c oxidase subunit 6A1, mitochondrial	10.0	L	21.0	M	2.15	OE

VL: very low; L: low; M: medium; H: high; UE: underexpressed; OE: overexpressed; NSAF: normalized spectral abundance factor; Abun: abundance; SC: spectral count.

**Table 3:** Differentially expressed proteins and their abundance in semen samples from group 1 (≥1 × 10^6^/mL leukocytes), group 3 (round cells < 1 × 10^6^/mL), and control group (pure leukocyte culture).

Uniprot No.	Gene Name	Protein Name	Group 1		Group 3		Control Group (CG)		NSAF Ratio CG/ Group1	Expression in CG
			SC	Abun	SC	Abun	SC	Abun		
P00352	ALDH1A1	Retinal dehydrogenase 1	26.3	M	2.7	VL	0.0	-	0.00	Absent in CG
Q6PEW0	PRSS54	Inactive serine protease 54	18.0	L	4.7	VL	0.0	-	0.00	Absent in CG
Q76KD6	SPATC1	Speriolin	8.3	L	2.3	VL	0.0	-	0.00	Absent in CG
Q9HAE3	EFCAB1	EF-hand calcium-binding domain-containing protein 1	9.3	L	3.3	VL	0.0	-	0.00	Absent in CG
O43242	PSMD3	26S proteasome non-ATPase regulatory subunit 3	33.7	M	78.3	M	0.0	-	0.00	Absent in CG
P23396	RPS3	40S ribosomal protein S3	24.7	M	60.0	M	0.0	-	0.00	Absent in CG
Q9NZM1-3	MYOF	Isoform 3 of Myoferlin	21.3	M	55.3	M	0.0	-	0.00	Absent in CG
Q96RQ1	ERGIC2	Endoplasmic reticulum-Golgi intermediate compartment protein 2	8.3	L	23.0	M	0.0	-	0.00	Absent in CG
Q8IYV9	IZUMO1	Izumo sperm-egg fusion protein 1	9.0	L	28.7	M	0.0	-	0.00	Absent in CG
Q04609	FOLH1	Glutamate carboxypeptidase 2	27.0	M	96.3	H	0.0	-	0.00	Absent in CG
Q8IY17-4	PNPLA6	Isoform 4 of Neuropathy target esterase	3.7	VL	14.0	L	0.0	-	0.00	Absent in CG
P62277	RPS13	40S ribosomal protein S13	5.3	VL	20.7	M	0.0	-	0.00	Absent in CG
Q13093	PLA2G7	Platelet-activating factor acetylhydrolase	5.3	VL	22.7	M	0.0	-	0.00	Absent in CG
O43653	PSCA	Prostate stem cell antigen	1.7	VL	14.3	L	0.0	-	0.00	Absent in CG
Q96M98-2	PACRG	Isoform 2 of Parkin coregulated gene protein	1.3	VL	21.0	M	0.0	-	0.00	Absent in CG
P46777	RPL5	60S ribosomal protein L5	5.7	VL	24.7	M	1.3	M	0.36	UE
P15289	ARSA	Arylsulfatase A	34.0	M	1.7	VL	4.7	VL	0.20	UE
P02788	LTF	Lactotransferrin	2486.7	H	1299.0	H	424.3	H	0.24	UE
P14314	PRKCSH	Glucosidase 2 subunit beta	123.0	H	65.0	M	33.7	M	0.39	UE
P27797	CALR	Calreticulin	271.7	H	175.0	H	123.0	H	0.65	UE
P14625	HSP90B1	Endoplasmin	987.0	H	660.3	H	73.3	M	0.11	UE
P05023-4	ATP1A1	Isoform 4 of Sodium/potassium-transporting ATPase subunit alpha-1	98.7	H	181.3	H	27.3	M	0.39	UE
O95202	LETM1	Mitochondrial proton/calcium exchanger protein	29.0	M	65.7	M	9.0	L	0.46	UE
Q9Y4W6	AFG3L2	AFG3-like protein 2	15.3	L	40.7	M	0.7	VL	0.06	UE
P11234	RALB	Ras-related protein Ral-B	9.0	L	22.0	M	62.0	M	10.12	OE
O43707	ACTN4	Alpha-actinin-4	44.7	M	23.3	L	52.3	M	1.73	OE

Expression VL: very low; L: low; M: medium; H: high; UE: underexpressed; OE: overexpressed; NSAF: normalized spectral abundance factor; Abun: abundance; SC: spectral count; CG: control group.

## Supplementary Materials

Supplementary materials can be found at http://www.mdpi.com/1422-0067/20/2/314/s1.
