# Supplementary material for: Presence of Round Cells Proteins do not Interfere with Identification of Human Sperm Proteins from Frozen Semen Samples by LC-MS/MS
Source: Int J Mol Sci. 2019 Jan 14;20(2):314. doi: 10.3390/ijms20020314 (PMC6359632; doi:10.3390/ijms20020314)
Supplement: Supplementary file 1 [file ijms-20-00314-s001.zip › Supplementary Files/Supplementary Table 1.doc]

| **Sample** | **Neat semen** | | | | | | | | **65% density gradient** | | | |
| --- | --- | --- | --- | --- | --- | --- | --- | --- | --- | --- | --- | --- |
|  | **Volume (mL)** | **pH** | **Color** | **Viscosity** | **Conc**  **(10^6^/mL)** | **Motility (%)** | **Round cells (10^6^/mL)** | **Endtz**  **(10^6^/mL)** | **Final Volume (mL)** | **Conc**  **(10^6^/mL)** | **Round cells (10^6^/mL)** | **Endtz**  **(10^6^/mL)** |
| D1 | 2.3 | 7.6 | Opaque | Normal | 57.00 | 72 | 1.20 | 1.2 | 1.0 | 50.40 | 0.0 | 0.0 |
| D2 | 4.0 | 7.6 | Opaque | Normal | 170.0 | 76 | 1.40 | 0.6 | 1.0 | 184.00 | 0.0 | 0.0 |
| D3 | 2.2 | 7.6 | Opaque | Normal | 59.00 | 76 | 1.20 | 1.2 | 1.0 | 48.00 | 0.0 | 0.0 |
| D4 | 1.8 | 7.6 | Opaque | Normal | 42.50 | 59 | 3.40 | 2.2 | 1.0 | 31.60 | 0.0 | 0.0 |
| D5 | 2.0 | 7.6 | Opaque | Normal | 69.00 | 86 | 1.60 | 1.3 | 1.0 | 40.00 | 0.0 | 0.0 |
| D6 | 3.6 | 7.6 | Opaque | Normal | 70.00 | 61 | 0.60 | N/A | 1.0 | 115.00 | 0.0 | 0.0 |
| D7 | 1.8 | 7.6 | Opaque | Normal | 55.50 | 82 | 0.10 | N/A | 1.0 | 43.80 | 0.0 | 0.0 |
| D8 | 5.5 | 7.6 | Opaque | Normal | 111.20 | 60 | 0.80 | N/A | 2.0 | 123.75 | 0.0 | 0.0 |
| D9 | 5.5 | 7.6 | Opaque | Normal | 15.30 | 66 | 0.40 | N/A | 1.0 | 31.00 | 0.0 | 0.0 |
| D10 | 2.5 | 7.6 | Opaque | Slight | 59.25 | 58 | 0.35 | N/A | 1.0 | 51.75 | 0.0 | 0.0 |

**Supplementary Table 1:** Semen parameters in normozoospermic healthy volunteers used for proteomic analysis
