# Supplementary material for: Presence of Round Cells Proteins do not Interfere with Identification of Human Sperm Proteins from Frozen Semen Samples by LC-MS/MS
Source: Int J Mol Sci. 2019 Jan 14;20(2):314. doi: 10.3390/ijms20020314 (PMC6359632; doi:10.3390/ijms20020314)
Supplement: Supplementary file 1 [file ijms-20-00314-s001.zip › Supplementary Files/Supplementary Table 2.doc]

**Supplementary Table 2:** Total number of proteins detected in group 1, 2, 3 and 4 run in triplicates (N1, N2 and N3), and differentially expressed proteins in group 1 vs 3, and group 2 vs 4.

| **Group** | **Proteins detected in each run** | | | **%CV** | **Total number of proteins** | **DEPs** | | | |
| --- | --- | --- | --- | --- | --- | --- | --- | --- | --- |
|  | **N1** | **N2** | **N3** |  |  | **Total** | **OE** | **UE** | **Unique** |
| 1 | 1543 | 1606 | 1579 | 2.01 | 1638 | 26 (1.18%) | 16 | 10 | - |
| 3 | 1647 | 1695 | 1643 | 1.75 | 1755 |  |  |  |  |
| 2 | 1445 | 1425 | 1579 | 4.01 | 1393 | 6 (0.31%) | 1 | 4 | 1 unique to group 2 |
| 4 | 1292 | 1401 | 1556 | 9.37 | 1404 |  |  |  |  |

CV: coefficient of variation, DEPs: differentially expressed proteins; OE: overexpressed, UE: underexpressed
